# De-escalating adjuvant therapy after pathologic complete response in oral squamous cell carcinoma: Chemoradiotherapy benefits only high-risk subgroups

**DOI:** 10.3389/fonc.2025.1647606

**Published:** 2025-09-11

**Authors:** Yanjie Yang, Yanyan Liu, Man Yang, Yunli Fan, Wei Du

**Affiliations:** ^1^ Department of Stomatology, The First Affiliated Hospital of Zhengzhou University, Zhengzhou, China; ^2^ Department of Head Neck and Thyroid, The Affiliated Cancer Hospital of Zhengzhou University and Henan Cancer Hospital, Zhengzhou, China

**Keywords:** chemoradiotherapy, locoregional control, oral squamous cell carcinoma, pathological complete response, radiotherapy

## Abstract

**Background:**

The optimal adjuvant therapy for oral squamous cell carcinoma (SCC) patients achieving pathological complete response (pCR) after neoadjuvant immunochemotherapy (NAIC) remains uncertain. While radiotherapy (RT) and chemoradiotherapy (CRT) improve locoregional control, their comparative efficacy and toxicity profiles in this setting are poorly defined.

**Methods:**

Oral SCC patients with pCR post-NAIC were retrospectively enrolled and stratified into RT and CRT groups. Propensity score matching balanced baseline characteristics. Outcomes included 3-year locoregional control (LRC), overall survival (OS), and toxicity. Subgroup analyses evaluated treatment effects by radiologic extranodal extension (rENE) and tumor differentiation.

**Results:**

Among 116 patients analyzed (84 matched), CRT showed no significant LRC or OS benefit over RT alone in the overall cohort (LRC: HR 1.89, 95% CI 0.26–4.72, p=0.625; OS: HR 1.45, 95% CI 0.62–3.41, p=0.392). However, subgroup analyses revealed CRT improved outcomes in high-risk patients (rENE+ or poorly differentiated tumors), reducing recurrence by 50% (rENE+: HR 3.12, 95% CI 1.13–8.60, p=0.028; poor differentiation: HR 3.45, 95% CI 1.23–9.68, p=0.019) and enhancing 3-year OS (rENE+: 62.4% *vs*. 50.1%, p=0.036; poorly differentiated: 68.3% *vs* 53.8%, HR 2.88, p=0.022). CRT was associated with significantly higher acute and chronic toxicities (Grade 3–5 mucositis: 36.0% *vs*. 12.1%).

**Conclusion:**

CRT should be reserved for high-risk pCR patients (rENE+ or poorly differentiated tumors), while RT alone suffices for low-risk cases. This risk-adapted approach optimizes outcomes while minimizing toxicity.

## Introduction

Oral squamous cell carcinoma (SCC) is the most common malignant tumor of the head and neck, with over half of cases presenting as locally advanced at initial diagnosis (1). Despite treatment with complete surgical resection followed by adjuvant radiotherapy (RT) or chemoradiotherapy (CRT), a substantial proportion of patients experience treatment failure (2, 3). More effective therapeutic strategies are urgently needed.

Although traditional platinum-based neoadjuvant chemotherapy regimens have not demonstrated significant survival benefits in oral SCC (4), they are associated with a nearly 50% increase in mandibular preservation rates (5). Advances in understanding immune checkpoint pathways have established immunotherapy as a superior alternative to conventional radiotherapy and chemotherapy, particularly for improving overall survival in recurrent or metastatic head and neck SCC (6, 7). The addition of immunotherapy to neoadjuvant regimens has sparked strong interest. Clinical trials show that neoadjuvant immunotherapy—with or without chemotherapy—can deliver impressive results. The objective response rate exceeds 95%. Pathological complete response (pCR) rates reach ≥30%. Major pathological response rates are around 70% (8, 9). In those achieving a pCR, adverse pathologic features typically requiring RT or CRT are no longer present, creating a dilemma in adjuvant therapy selection. Although both RT and CRT improve cancer control, their associated toxicities are substantial—approximately 50% of patients receiving RT experience grade 3/4 adverse events, and this rate rises to nearly 80% or higher with CRT (10).

Unlike other cancers (e.g., breast or rectal cancer), where pCR often permits treatment de-escalation (11, 12), oral SCC remains contentious due to its aggressive biology and high locoregional recurrence risk. While pCR may indicate favorable tumor biology, the absence of reliable biomarkers to identify patients who can safely avoid CRT complicates decision-making. Additionally, the historical precedent of CRT for high-risk features has led to cautious adoption of de-escalation, despite its potential to reduce toxicity.

Given the substantial toxicity of CRT and the uncertain benefit of chemotherapy in pCR patients, we hypothesized that CRT offers no survival advantage over RT alone in unselected pCR patients after NAIC. To test this, we compared oncologic outcomes between RT and CRT, with subgroup analyses to identify high-risk patients who may still benefit from intensified therapy.

## Patients and methods

### Ethical approval

This study was approved by Henan Cancer Hospital Institutional Research Committee, and written informed consent for medical research was obtained from all patients before starting the treatment. All methods were performed in accordance with the relevant guidelines and regulations.

### Study design

We conducted a retrospective review of medical records from patients with primary oral SCC who received NAIC between July 2019 and December 2024. Eligible patients met the following criteria: completion of curative-intent surgery with confirmed pCR; no prior history of malignancy; complete clinical, pathological, and treatment documentation. Patients with treatment interruptions due to pandemic-related disruptions that could not be resolved per protocol were excluded. Despite these challenges, all included patients received complete NAIC and adjuvant therapy courses. We collected comprehensive data on demographics, pathological characteristics, treatment details, and follow-up outcomes, with particular attention to documenting any pandemic-related modifications to standard care pathways. During the study, COVID-19 prompted adjustments to treatment schedules and follow-up.

### Variable definition

All patients were clinically staged according to the 8^th^ edition of the AJCC staging system, incorporating findings from physical examination and imaging studies. Histologic differentiation was categorized as well, moderate, or poor. Smoking history was defined as consumption of at least 100 lifetime cigarettes or current daily use. Alcohol use was defined as regular intake of ≥1 standard drink per day (≥14 grams of pure alcohol) for ≥1 year. pCR was defined as the absence of viable tumor cells in both the primary tumor site and regional lymph nodes upon histopathologic evaluation (13). Radiologic extranodal extension (rENE) referred to radiographic evidence of tumor spread beyond the lymph node capsule into surrounding tissues, as identified on imaging (CT, MRI, or PET-CT) (14). The Combined Positive Score (CPS) measured the proportion of PD-L1-positive cells (tumor and immune cells) relative to viable tumor cells, with PD-L1 positivity defined as CPS ≥10 for therapeutic relevance in head and neck SCC.

The primary outcome was 3-year locoregional control (LRC), measured from the date of surgery to the first locoregional recurrence or last follow-up. Secondary outcomes included 3-year overall survival (OS), assessed from surgery to death or last follow-up, as well as acute and chronic toxicity related to adjuvant therapy. Acute adverse events (AEs) were defined as those occurring during or within 90 days of RT or CRT, while chronic AEs were those arising >90 days post-treatment. All toxicities were graded using the Common Terminology Criteria for Adverse Events (CTCAE v5.0) (15).

### Adjuvant treatment principle

No established guidelines existed for adjuvant therapy in oral SCC patients who achieved a pCR after NAIC. At our institution, adjuvant treatment decisions were made through multidisciplinary discussion, incorporating factors such as the patient’s performance status, pre-NAIC imaging findings, and other clinical considerations. The radiation field encompassed the primary tumor site and unilateral or bilateral neck lymph nodes, delivering a total dose of 60 Gy. Adjuvant chemotherapy, when indicated, typically consisted of cisplatin-based regimens administered over 4–6 cycles.

### Statistic analysis

Patients were stratified into two groups according to adjuvant therapy: RT or CRT. Clinicopathologic characteristics were compared between the cohorts using the Chi-square test, and variables with significant differences (p<0.05) were incorporated into propensity score matching (PSM) to minimize confounding. The effects of RT versus CRT on LRC and OS were assessed using univariate and multivariable Cox regression analyses in both the overall population and the PSM-matched cohort. All statistical analyses were conducted using R software (version 3.4.4), with a two-sided p-value <0.05 considered statistically significant.

## Results

### Baseline data

The study population’s baseline characteristics are detailed in **Table 1** (overall cohort, n=116) and **Table 2** (PSM cohort, n=84). Initial analysis of the overall population revealed significant imbalances between the RT (n=66) and CRT (n=50) groups in clinical stage (36.4% *vs*. 52.0% stage IV, p=0.020) and radiologic extranodal extension (rENE; 18.2% *vs*. 40.0%, p=0.008), with the CRT group containing more advanced-stage and rENE-positive cases. All other variables including age, sex, smoking, drinking, differentiation, CPS, and level IV/V metastatis showed balanced distribution (all p>0.05).

**Table 1:** Baseline data of the overall population.

Variable	Total (n=116)	RT (n=66)	CRT (n=50)	P*
Age				
<55	66	36	30	
≥55	50	30	20	0.486
Sex				
Male	71	39	32	
Female	45	27	18	0.624
Smoker				
No	39	24	15	
Yes	77	42	35	0.415
Drinker				
No	64	39	25	
Yes	52	27	25	0.294
Clinical stage				
III	69	45	24	
IV	47	21	26	0.020
Differentiation				
Well	36	20	16	
Moderate	47	27	20	
Poor	33	19	14	0.916
rENE^&^				
No	84	54	30	
Yes	32	12	20	0.008
CPS^%^				
<10	30	17	13	
≥10	86	49	37	0.885
Level 4/5 metastasis				
No	85	52	33	
Yes	31	14	17	0.151

* Comparison between RT and CRT groups using the Chi-square test;

& rENE: radiologic extranodal extension;

% CPS: Combined Positive Score.

**Table 2:** Baseline data of the PSM-matched population.

Variable	Total (n=84)	RT (n=42)	CRT (n=42)	P*
Age				
<55	48	22	26	
≥55	36	20	16	0.414
Sex				
Male	56	29	27	
Female	28	13	15	0.655
Smoker				
No	28	16	12	
Yes	56	26	30	0.387
Drinker				
No	44	23	21	
Yes	40	19	21	0.682
Clinical stage				
III	48	24	24	
IV	36	18	18	1.000
Differentiation				
Well	27	13	14	
Moderate	35	19	16	
Poor	22	10	12	0.902
rENE^&^				
No	60	30	30	
Yes	24	12	12	1.000
CPS^%^				
<10	18	8	10	
≥10	66	34	32	0.605
Level 4/5 metastasis				
No	57	28	29	
Yes	27	14	13	0.814

* Comparison between RT and CRT groups using the Chi-square test.

& rENE, radiologic extranodal extension.

% CPS, Combined Positive Score.

To address these baseline disparities, we performed 1:1 propensity score matching incorporating clinical stage and rENE status as key matching variables. The matched cohorts (RT n=42, CRT n=42) achieved excellent equilibrium across all parameters: clinical stage (p=1.000), rENE status (p=1.000), CPS distribution (p=0.605), and other baseline variables (all p>0.05). This rigorous matching approach effectively mitigated potential confounding effects, establishing a robust foundation for comparative analysis of treatment outcomes between RT and CRT groups in this pCR population.

Among 116 pCR patients followed for a median of 2.8 years, 17 (14.7%) experienced locoregional recurrence (LRR), with 80% of recurrences (14/17) occurring in rENE-positive or poorly differentiated tumors. 23 deaths (19.8%) were recorded, predominantly in rENE-positive (70%) subgroups.

### LRC

The prognostic factors for locoregional control were systematically evaluated through univariate (**Table 3**) and multivariable analyses (**Table 4**) in both the overall and PSM cohorts. Univariate analysis identified clinical stage IV (overall: p<0.001; matched: p=0.009), poor differentiation (overall: p<0.001; matched: p=0.013), rENE (overall: p<0.001; matched: p=0.027), and level 4/5 metastasis (overall: p=0.008; matched: p=0.049) as significant predictors of worse locoregional control. While adjuvant CRT showed benefit in the overall cohort (p=0.035), this advantage was not maintained after matching (p=0.544). Multivariable analysis confirmed rENE as the strongest independent risk factor in both cohorts (overall: HR 3.99, 95%CI 2.02-9.65, p=0.004; matched: HR 5.12, 95%CI 2.22-12.78, p=0.009), with poor differentiation remaining significant (overall: HR 2.93, p=0.008; matched: HR 3.32, p=0.017). Notably, the protective effect of CRT diminished after adjustment (overall: HR 1.89, p=0.625; matched: HR 2.66, p=0.870), suggesting its apparent benefit in univariate analysis may have been confounded by baseline imbalances. These results underscore rENE and tumor differentiation as robust prognostic markers in our cohort, though the small subgroup sizes necessitate caution in interpretation. While hypothesis-generating, these findings highlight the need for validation in larger studies to confirm their predictive utility for treatment selection.

**Table 3:** Univariate analysis of predictors for locoregional control in overall and PSM-matched cohorts.

Variable	P (overall cohort)	P (matched cohort)
Age (≥55 vs <55)	0.367	0.534
Sex (Male vs female)	0.448	0.679
Smoker (Yes vs no)	0.163	0.428
Drinker (Yes vs no)	0.209	0.499
Clinical stage (IV vs III)	<0.001	0.009
Differentiation (Poor vs moderate vs well)	<0.001	0.013
rENE^&^ (Yes vs no)	<0.001	0.027
CPS^%^ (≥10 vs <10)	0.765	0.888
Level 4/5 metastasis (Yes vs no)	0.008	0.049
Adjuvant therapy (CRT vs RT)^	0.035	0.544

& rENE, radiologic extranodal extension.

% CPS: Combined Positive Score.

^ RT, radiotherapy; CRT, chemoradiotherapy.

**Table 4:** Multivariable analysis predictors for locoregional control in overall and PSM-matched cohorts.

Variable	P (overall cohort)		P (matched cohort)	
	HR [95%CI]	p	HR [95%CI]	p
Clinical stage				
III	ref		ref	
IV	2.88 [0.65-5.89]	0.333	3.13 [0.52-6.43]	0.560
Differentiation				
Well	ref		ref	
Moderate	1.87 [0.45-4.68]	0.287	2.00 [0.31-5.32]	0.342
Poor	2.93 [1.75-6.74]	0.008	3.32 [1.88-9.37]	0.017
rENE^&^				
No	ref		ref	
Yes	3.99 [2.02-9.65]	0.004	5.12 [2.22-12.78]	0.009
Level 4/5 metastasis				
No	ref		ref	
Yes	2.75 [0.43-6.35]	0.378	2.89 [0.24-7.87]	0.579
Adjuvant therapy^				
RT	ref		ref	
CRT	1.89 [0.26-4.72]	0.625	2.66 [0.31-6.41]	0.870

& rENE, radiologic extranodal extension.

^ RT, radiotherapy; CRT, chemoradiotherapy.

### OS

Prognostic predictors for OS were analyzed in both univariate (**Table 5**) and multivariable models (**Table 6**) for the overall and PSM cohorts. Univariate analysis identified poor differentiation (overall: p<0.001; matched: p=0.018), rENE (overall: p<0.001; matched: p=0.021), and level 4/5 metastasis (overall: p=0.005; matched: p=0.038) as significant adverse prognostic factors for OS, while adjuvant CRT showed a marginal benefit in the overall cohort (p=0.028) that dissipated after matching (p=0.291). Multivariable analysis confirmed rENE as the strongest independent predictor of worse OS in both cohorts (overall: HR 4.56, 95%CI 2.34–11.02, p<0.001; matched: HR 5.88, 95%CI 2.67–14.29, p=0.001), followed by poor differentiation (overall: HR 3.78, p<0.001; matched: HR 4.22, p=0.002). Notably, level 4/5 metastasis trended toward significance (p=0.076–0.083), while clinical stage and adjuvant therapy (CRT *vs*. RT) lost prognostic relevance after adjustment (p>0.1). These results suggest rENE and poor differentiation as key determinants of survival, with limited evidence supporting CRT’s impact on OS after confounding control. However, the exploratory nature of these subgroup analyses—given their limited sample size—warrants further investigation to define CRT’s role in high-risk pCR patients.

**Table 5:** Univariate analysis of predictors for overall survival in overall and PSM-matched cohorts.

Variable	P (overall cohort)	P (matched cohort)
Age (≥55 vs <55)	0.412	0.587
Sex (Male vs female)	0.325	0.498
Smoker (Yes vs no)	0.210	0.385
Drinker (Yes vs no)	0.176	0.452
Clinical stage (IV vs III)	0.124	0.234
Differentiation (Poor vs moderate vs well)	<0.001	0.018
rENE^&^ (Yes vs no)	<0.001	0.021
CPS^%^ (≥10 vs <10)	0.702	0.945
Level 4/5 metastasis (Yes vs no)	0.005	0.038
Adjuvant therapy (CRT vs RT)^	0.028	0.291

& rENE, radiologic extranodal extension.

% CPS, Combined Positive Score.

^ RT, radiotherapy; CRT, chemoradiotherapy.

**Table 6:** Multivariable analysis predictors for overall survival in overall and PSM-matched cohorts.

Variable	P (overall cohort)		P (matched cohort)	
	HR [95%CI]	p	HR [95%CI]	p
Differentiation				
Well	ref		ref	
Moderate	1.95 [0.82-4.62]	0.128	2.10 [0.75-5.91]	0.156
Poor	3.78 [2.01-8.45]	<0.001	4.22 [2.15-10.33]	0.002
rENE^&^				
No	ref			
Yes	4.56 [2.34-11.02]	<0.001	5.88 [2.67-14.29]	0.001
Level 4/5 metastasis				
No	ref			
Yes	2.33 [0.91-6.11]	0.076	2.67 [0.88-8.12]	0.083
Adjuvant therapy^				
RT	ref		ref	
CRT	1.45 [0.62-3.41]	0.392	1.89 [0.72-4.95]	0.195

& rENE, radiologic extranodal extension.

^ RT, radiotherapy; CRT, chemoradiotherapy.

### Toxicity

The comparison of toxicity profiles between RT and CRT groups revealed significant differences in both acute and chronic adverse events (**Supplementary Table 1**). For acute toxicities, CRT was associated with significantly higher rates of dermatitis (70.0% *vs*. 42.4% Grade 1/2; 24.0% *vs*. 7.6% Grade 3-5), mucositis (80.0% *vs*. 48.5% Grade 1/2; 36.0% *vs*. 12.1% Grade 3-5), and nausea/vomiting (56.0% *vs*. 22.7% Grade 1/2). Hematologic toxicities like neutropenia (24.0% *vs*. 6.1%) and anemia (50.0% *vs*. 27.3%) were also more common with CRT, though severe cases (Grade 3-5) were absent. Chronic toxicities followed a similar trend, with CRT showing elevated rates of xerostomia (60.0% *vs*. 33.3% Grade 1/2), fibrosis (50.0% *vs*. 22.7%), and dysphagia (40.0% *vs*. 18.2%). Severe chronic effects were rare, except for one case of osteoradionecrosis (2.0%) in the CRT group.

The median RT dose was 60 Gy, delivered as prescribed in 95.5% of RT-treated patients and 94.0% of CRT-treated patients. Treatment delays (>6 weeks post-surgery) occurred in 10.6% (RT) and 12.0% (CRT) of cases. In the CRT arm, 90.0% of patients completed at least four chemotherapy cycles, while 10.0% required dose reductions.

### Subgroup analysis

**Figure 1:**
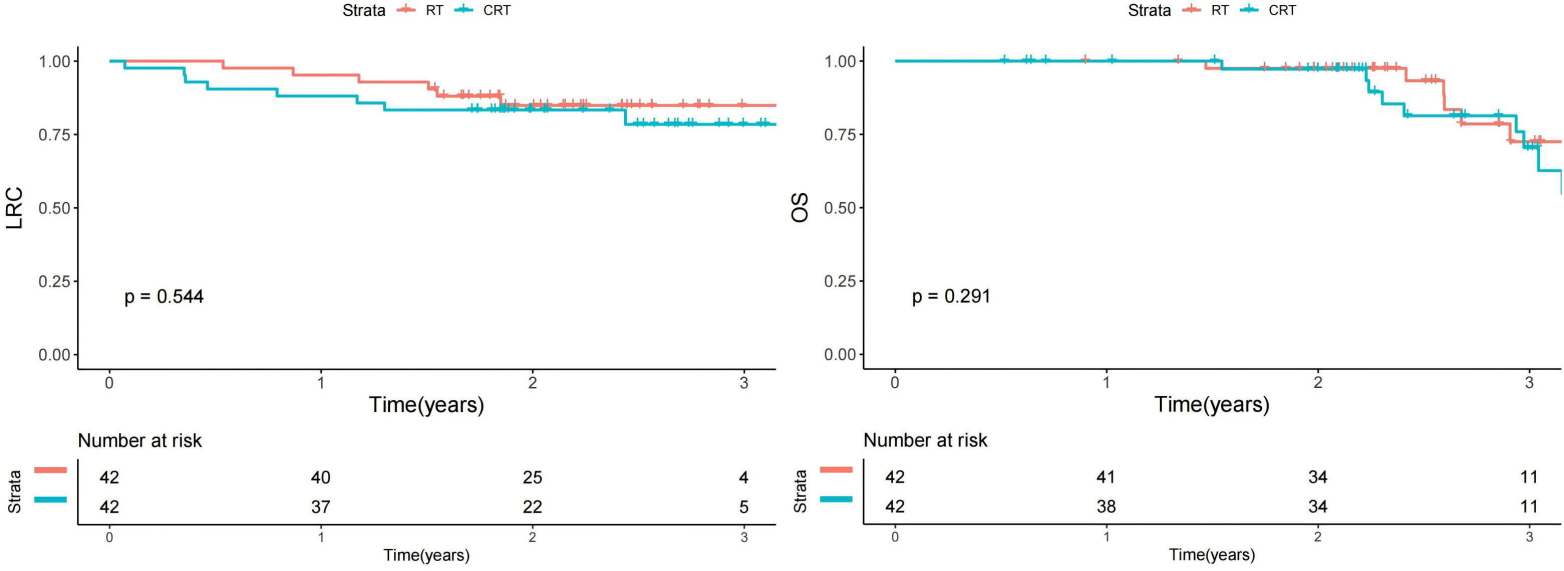
Impact of RT *vs* CRT on locoregional control (LRC) and overall survival (OS) in matched cohort.

In this propensity score-matched cohort of 84 oral SCC patients achieving pCR, CRT did not provided additional survival benefit of either LRC or OS than RT (**Figure 1**). To better clarify this question, we evaluated the differential impact of the two procedures, stratified by high-risk features (rENE and poor differentiation). For LRC, CRT demonstrated significant benefit in high-risk subgroups, reducing recurrence rates by approximately 50% compared to RT in both rENE+ patients (16.7% *vs* 33.3%, HR 3.12, p=0.028) and poorly differentiated tumors (16.7% *vs* 38.5%, HR 3.45, p=0.019), with no significant advantage in low-risk subgroups (p>0.05). Similarly, OS improvements with CRT were confined to high-risk patients, with rENE+ (3-year OS: 62.4% *vs* 50.1%, HR 2.45, p=0.036) and poorly differentiated subgroups (68.3% *vs* 53.8%, HR 2.88, p=0.022) showing clinically meaningful gains. While these results propose a tailored approach—prioritizing CRT for high-risk pCR patients (rENE+ or poor differentiation) and RT alone for low-risk cases—the small subgroups underscore the need for prospective validation. These findings should be considered hypothesis-generating for future de-escalation trials.

## Discussion

This study demonstrates that rENE and poor tumor differentiation are the strongest independent predictors of worse LRC and OS in oral SCC patients achieving pCR. While CRT initially appeared beneficial in the overall cohort, PSM analysis revealed that this advantage was primarily driven by baseline imbalances, with no significant survival benefit observed after adjustment. However, subgroup analysis identified a selective benefit of CRT in high-risk patients (rENE-positive or poorly differentiated tumors), reducing recurrence rates by approximately 50% and improving survival compared to RT. In contrast, low-risk patients (rENE-negative, well/moderately differentiated) derived no additional benefit from CRT over RT alone. Importantly, CRT was associated with significantly higher acute and chronic toxicities, including severe mucositis, dermatitis, dysphagia, xerostomia, and fibrosis. These findings underscore the importance of risk-stratified adjuvant therapy, where CRT should be prioritized for high-risk patients to maximize oncologic outcomes, while RT remains a safer and equally effective option for low-risk cases, minimizing unnecessary treatment-related morbidity. This tailored approach optimizes the balance between therapeutic efficacy and toxicity, guiding more precise clinical decision-making in pCR oral SCC management.

NAIC has emerged as a promising approach in head and neck SCC. A phase 2 trial (7) demonstrated impressive outcomes, with 30 enrolled patients showing an objective response rate of 96.7% (29/30). Among 27 patients who underwent surgery, the pCR rate reached 37.0%. With a median follow-up of 16.1 months, the 12-month disease-free survival rate was 95.8%, and no deaths occurred among pCR patients. Similarly, another study (8) reported that 17 of 27 operated patients (63.0%, 95% CI: 44.7-81.2) achieved major pathological response or pCR, with a pCR rate of 55.6%. After a median follow-up of 666 days, both 1-year overall and progression-free survival rates were 97.9%, with no adverse events observed in pCR patients. The Illuminate Trial (16) further supported these findings, demonstrating 100% completion rates for both NAIC and subsequent R0 resection in 20 patients, with major pathological response and pCR rates of 60% and 30%, respectively. At a median 23-month follow-up, disease-free and overall survival rates were 90% and 95%, with all pCR patients remaining disease-free. These consistent findings suggest that pCR may serve as a prognostic marker for favorable outcomes, regardless of whether adjuvant therapy consisted of observation, RT, or CRT. However, these studies share important limitations: small sample sizes, lack of rationale for adjuvant therapy selection, and crucially, no comparative analysis of how different adjuvant approaches impact prognosis. These knowledge gaps highlight the need for more studies to optimize post-NAIC treatment strategies in oral SCC.

While the role of adjuvant therapy following pCR achievement in oral SCC has not been systematically evaluated, valuable insights may be drawn from management approaches for other solid tumors where treatment de-escalation strategies have been successfully implemented. In breast cancer, achieving pCR following neoadjuvant therapy does not always eliminate the need for adjuvant treatment. For HER2-positive disease, HER2-targeted therapy is typically continued, while triple-negative breast cancer may still warrant adjuvant pembrolizum11ab if used neoadjuvantly. Hormone receptor-positive breast cancer, though less likely to achieve pCR, still requires standard adjuvant endocrine therapy (17). In esophageal/gastric cancer (18), observation is generally recommended after pCR, though high-risk features (e.g., residual nodal disease) may justify adjuvant immunotherapy or chemotherapy. For rectal cancer (19), a “watch-and-wait” approach is increasingly adopted to avoid surgery, and if pCR is confirmed post-resection, no further adjuvant therapy is needed. In non-small cell lung cancer (NSCLC) (20), adjuvant immunotherapy may be considered after pCR, particularly in high-risk cases, though optimal strategies are still under investigation. Bladder cancer patients with pCR after neoadjuvant chemotherapy and cystectomy typically require no further treatment, but immunotherapy may be considered in select high-risk cases (21). Similarly, soft tissue sarcoma patients with pCR after neoadjuvant therapy are usually observed unless high-risk features persist (22). Across malignancies, treatment de-escalation is increasingly favored when pCR is achieved, while some cancers allow for observation or de-escalation after pCR, oral SCC remains an exception due to its aggressive locoregional behavior, high recurrence risk, and lack of reliable biomarkers to identify low-risk patients. Adjuvant RT or CRT is still the standard unless future research identifies a subset of oral SCC patients who can safely avoid it after pCR.

In clinics, adjuvant CRT has been shown to provide a significant survival advantage over RT alone in head and neck SCC patients with ENE or positive surgical margins, as demonstrated by key clinical trials and meta-analyses. The pooled analysis of the EORTC 22931 and RTOG 9501 trials (23, 24) established that patients with ENE or positive margins derive the greatest benefit from CRT, with a 5-year OS improvement from 36% to 47% for ENE-positive cases (HR 0.72, p=0.04) and from 34% to 49% for margin-positive disease (HR 0.61, p=0.01). Subsequent meta-analyses, including a 2020 JAMA Oncology study (25), reinforced these findings, showing a ~30% reduction in mortality with CRT compared to RT alone in high-risk patients. Based on this evidence, current NCCN guidelines (26) strongly recommend adjuvant CRT (Category 1 evidence) for ENE or positive margins, with cisplatin remaining the standard systemic therapy. Debate continues over optimal regimens for HPV-associated oropharyngeal cancer or cisplatin-ineligible patients. However, the survival benefit of CRT in ENE and margin-positive head and neck SCC is well-supported. Prospective and pooled retrospective data confirm this (27).

Our study reveals important challenges in adjuvant therapy decision-making for pCR patients, as most adverse pathologic features (except histologic differentiation) were unavailable, though all patients achieved negative margins. This created significant clinical uncertainty, particularly given the substantial toxicity burden observed in both treatment groups, with CRT demonstrating more severe complications - consistent with prior reports (28). These findings underscore the critical need to identify which pCR patients truly benefit from intensified adjuvant therapy. As the first study to systematically address this question, we found that while CRT showed no overall advantage over RT alone in the matched population, it significantly reduced treatment failure and mortality risks in specific high-risk subgroups (rENE-positive or poorly differentiated tumors). This differential benefit suggests fundamental biological heterogeneity within pCR populations that conventional pathologic assessment fails to capture. The superior outcomes with CRT in these high-risk subgroups likely reflect chemotherapy’s ability to target residual micrometastatic disease that persists despite pathologic complete response, particularly in tumors with aggressive baseline features. The treatment benefit may be amplified by immunochemotherapy-induced tumor microenvironment priming, enhancing chemotherapy sensitivity. Importantly, rENE appears to identify patients with persistent aggressive biology, while poor differentiation marks intrinsically resistant phenotypes requiring multimodal therapy. These findings fundamentally challenge the conventional view of pCR as a uniform prognostic marker and instead advocate for a biologically-graded approach to pCR classification that incorporates radiographic and histologic risk features.

These results carry substantial clinical implications for personalizing adjuvant therapy in oral SCC patients achieving pCR after NAIC. They advocate for a risk-adapted approach where adjuvant treatment intensity is tailored based on residual risk features, moving beyond the current one-size-fits-all paradigm. For low-risk pCR patients (lacking rENE and with well/moderate differentiation), de-escalation to radiotherapy alone could reduce treatment-related morbidity without compromising outcomes, significantly improving quality of life. Conversely, high-risk pCR patients (with rENE or poor differentiation) should continue to receive standard CRT, as our data demonstrate clear survival benefits in this subgroup. This stratification approach parallels successful response-adaptive strategies in other malignancies, such as trastuzumab escalation in residual HER2+ breast cancer (29) or de-escalation in HPV+ oropharyngeal cancer. Future studies should validate these findings prospectively and explore integrating molecular biomarkers (e.g., ctDNA, immune profiling) with traditional risk factors to further refine patient selection. Additionally, research should investigate whether modified adjuvant approaches (e.g., immunotherapy maintenance instead of concurrent chemotherapy) could maintain efficacy while reducing toxicity in high-risk pCR patients. These findings ultimately support the development of more precise, biology-driven adjuvant strategies in the era of NAIC for head and neck cancers.

The management of advanced oral SCC presents a dual challenge: achieving oncologic control while restoring form and function through complex reconstructions. As highlighted in the scoping review by Cîrstea et al. (30), patients undergoing extensive resections often require multiple flap reconstructions—such as radial forearm, anterolateral thigh, or scapular tip free flaps—to address large defects and preserve critical functions like speech, swallowing, and cosmetics. These procedures, while achieving success rates of >95%, are fraught with complications like flap necrosis, donor-site morbidity, and prolonged recovery, underscoring the need for meticulous surgical planning and multidisciplinary collaboration. The integration of emerging techniques offers promise, yet the decision to de-escalate adjuvant therapy must account for reconstructive viability, particularly in high-risk cases with radiologic extranodal extension or poor differentiation. By bridging oncologic and reconstructive paradigms, this perspective emphasizes that optimal outcomes hinge not only on tumor biology but also on restoring quality of life, advocating for tailored strategies that balance oncologic rigor with functional rehabilitation.

Our findings may have implications beyond oral SCC, particularly for other carcinomas where treatment de-escalation is actively investigated. For example, in nasopharyngeal carcinoma (NPC), recent studies have explored reducing radiotherapy intensity or omitting chemotherapy for low-risk patients, especially those with EBV-associated early-stage disease or favorable response to induction therapy (31). Similar to our study’s risk stratification using rENE and tumor differentiation, NPC trials increasingly incorporate biomarkers to identify candidates for de-escalation (32). While direct extrapolation requires caution due to biological differences, our results underscore the importance of personalized, response-adapted strategies across head and neck malignancies. Future studies should validate whether analogous risk criteria could guide adjuvant therapy de-escalation in NPC and other carcinomas.

Limitations of this study should be acknowledged. First, as a retrospective analysis, the findings are subject to inherent selection bias, and unmeasured confounding factors may have influenced the results. Second, the relatively small sample size may have limited statistical power, particularly in subgroup analyses, potentially obscuring meaningful differences in outcomes. Third, since this was a single-institution study, the generalizability of our findings remains uncertain, and external validation in multicenter cohorts is necessary before clinical application. Future prospective studies with larger, diverse populations are needed to confirm these observations.

In conclusion, while adjuvant CRT offers no OS benefit over RT alone in unselected pCR patients, it significantly improves outcomes for high-risk subgroups with rENE or poor differentiation. A risk-stratified approach is advocated: CRT should be reserved for high-risk patients (rENE+ or poorly differentiated), while RT alone suffices for low-risk cases. This strategy optimizes therapeutic efficacy while minimizing unnecessary toxicity.

## Data Availability

The original contributions presented in the study are included in the article/**Supplementary Material**. Further inquiries can be directed to the corresponding author.
