# Supplementary material for: De-escalating adjuvant therapy after pathologic complete response in oral squamous cell carcinoma: Chemoradiotherapy benefits only high-risk subgroups
Source: Front Oncol. 2025 Sep 11;15:1647606. doi: 10.3389/fonc.2025.1647606 (PMC12460139; doi:10.3389/fonc.2025.1647606)
Supplement: Supplementary file 1 [file Table1.doc]

Supplementary Table 1. Acute and chronic toxicity in the overall population treated by RT or CRT.

| **Acute** | Grade 1/2 | | Grade 3-5 | |
| --- | --- | --- | --- | --- |
|  | RT | CRT | RT | CRT |
| Dermatitis | 28 (42.4%) | 35 (70.0%) | 5 (7.6%) | 12 (24.0%) |
| Mucositis | 32 (48.5%) | 40 (80.0%) | 8 (12.1%) | 18 (36.0%) |
| Xerostomia | 45 (68.2%) | 38 (76.0%) | 2 (3.0%) | 4 (8.0%) |
| Dysphagia | 30 (45.5%) | 32 (64.0%) | 2 (3.0%) | 5 (10.0%) |
| Neutropenia | 4 (6.1%) | 12 (24.0%) | 0 | 0 |
| Anemia | 18 (27.3%) | 25 (50.0%) | 0 | 0 |
| Thrombocytopenia | 5 (7.6%) | 10 (20.0%) | 0 | 0 |
| Nausea/vomiting | 15 (22.7%) | 28 (56.0%) | 1 (1.5%) | 2 (4.0%) |
| Fatigue | 25 (37.9%) | 28 (56.0%) | 0 | 0 |
| Anorexia | 20 (30.3%) | 30 (60.0%) | 0 | 0 |
| Infection | 6 (9.1%) | 14 (28.0%) | 0 | 0 |
|  |  |  |  |  |
| **Chronic** | Grade 1/2 | | Grade 3-5 | |
|  | RT | CRT | RT | CRT |
| Xerostomia | 22 (33.3%) | 30 (60.0%) | 1 (1.5%) | 2 (4.0%) |
| Fibrosis | 15 (22.7%) | 25 (50.0%) | 0 | 0 |
| Dysphagia | 12 (18.2%) | 20 (40.0%) | 0 | 0 |
| Ototoxicity | 5 (7.6%) | 10 (20.0%) | 0 | 0 |
| Hypothyroidism | 8 (12.1%) | 14 (28.0%) | 0 | 0 |
| Trismus | 10 (15.2%) | 18 (36.0%) | 0 | 0 |
| Lymphedema | 6 (9.1%) | 12 (24.0%) | 0 | 0 |
| Neuropathy | 3 (4.5%) | 8 (16.0%) | 0 | 0 |
| Osteoradionecrosis | 0 | 0 | 0 | 1 (2.0%) |
| Dental caries | 9 (13.6%) | 16 (32.0%) | 0 | 0 |
